# A Generic Model to Assess the Efficiency Analysis of Cellular Foams

**DOI:** 10.3390/ma17030746

**Published:** 2024-02-04

**Authors:** Massimiliano Avalle

**Affiliations:** Dipartimento di Ingegneria Meccanica, Energetica, Gestionale e dei Trasporti, Università Degli Studi di Genova, Via All’Opera Pia, 15/A, 16145 Genova, Italy; massimiliano.avalle@unige.it

**Keywords:** cellular materials, energy diagrams, efficiency diagrams

## Abstract

One of the main types of uses of cellular materials is for energy absorption and dissipation in applications, such as safety and packaging, to protect people and goods during impact situations. In such cases, the use of cellular materials is justified by their capacity to largely deform under limited loads. This is often achieved, alone or within energy absorbing structures, with the additional advantage of cheap components that are relatively simple to manufacture and assemble. As in most engineering applications, weight reduction is sought after and, as in the case of other materials, this objective can be attained by optimizing the use of the material. Optimization of a cellular material for energy absorption means obtaining an optimal mechanical characteristic that can be obtained by properly designing it in terms of the type of base material and cell properties. Cell properties are mainly related to density and their optimal selection can be made by means of energy criteria. The aim of the present paper is to discuss such optimality criteria based on what are termed efficiency diagrams to produce an effective design tool. Additionally, based on empiric observations on the behavior of several classes of polymeric foams, a simplified selection method is proposed to hasten the selection criteria.

## 1. Introduction

Cellular materials [1] are used for several engineering applications including: insulation, mainly thermal; filtration; buoyancy; support (in seats and upholstery); and others. Among these other applications, cellular materials or foams are used for energy management through the absorption and dissipation of the initial kinetic energy of an impact to protect people and goods. These applications include packaging and vehicle safety. The use of cellular materials for these applications is related to the great range of compressive deformation that can be obtained with relatively low values of applied stress. Thus, the initial kinetic energy of an impact can be stored while maintaining the load transmitted to the protected items at levels compatible with the safety of the protected items themselves. The design of a component made of a cellular material typically starts with the selection of the solid material (this initial choice depends on various technical requirements such as, for example, operating temperature, compatibility with other components, recyclability, etc., as well as economical constraints) and then proceeds with the definition of the expansion grade; that is, the foam density. The optimal density for a specific application can be defined by using energetic principles implemented in methods based on efficiency parameters [2] or on cushioning curves. The use of efficiency curves represents a simpler and more effective method to optimize the energy absorption of a foam compared to using energy curves. The maximum efficiency identifies the optimal foam giving maximum absorbed energy for a certain maximum load level or, conversely, the minimum load for a certain value of absorbed energy. This method is useful in most applications and has been applied to several classical materials, including metal foams such as magnesium-based [3], aluminum-based [4], and titanium-based foams [5], as well as with innovative solutions such microcellular porous solids [6], density-graded polymeric [7] foams, polymeric [8] and aluminum honeycombs [9], lattice structures [10], auxetic structures [11], and open-cell lightweight structures [12]. The cushioning curves, instead, relate the maximum acceleration—defined as the G-value—in an impact to the stress: in the end, this G-value is simply the inverse of the efficiency. This method was proposed several decades ago and is defined by the international standard ISO 2248:1985 [13]. The method is examined in several papers: see [14], where the method is critically examined, and [15], where an efficient method to obtain the cushioning curve is proposed. Several papers include relevant data for materials widely used in packaging such as EPS [16] and PU [17].

Both these methods require the extensive experimental characterization of an expanded material by testing samples of the same solid material expanded at different grades (see [18], where the efficiency curves method was applied to the design of an energy absorber for worker security ropes; ref. [19], about the use of foams in sport applications; and [20], where the cushioning method was applied to an EPE energy absorber), and subsequent complex analysis of the results. The study of this requires testing under different loading conditions such as at different values of the strain rate [14,20]. It is a complex and expensive task that can be simplified by using predictive models of the expanded foam [15]. A recent interesting review soundly remarking on the importance of modeling in the design of foams was published by Rahimidehgolan and Altenhof in [21]. For the cushioning method, the effective use of modeling techniques is shown in [22,23].

In this paper, based on an empirical model by the same author [24], it will be shown how the design process can be simplified. By using a mathematical—though simplified and empirical—model, it is possible to predict the stress–strain, and then the absorbed energy and efficiency of a foam obtained from a solid material for different values of strain, strain rate, density and, possibly, temperature, etc. The method is explained and detailed with results for several polymeric foams, but it can also be applied to metal foams or honeycombs. Additionally, it will be shown that the method can be simplified even more based on a particular occurrence observed in the examined materials; providing a very quick method for approximately drawing selection curves for foams. This method can be considered as a valuable and effective tool for the preliminary design of energy-absorbing components where expanded materials are the main active subsystem. Of course, the simple analysis of the stress–strain characteristics of the foam, or of the energy diagram, is not sufficient for a definitive selection of either the class of materials nor the expansion level, and thus the foam density: several other factors have to be taken into account in a practical application (including durability, energy dissipation or elasticity versus viscoelasticity, and short-term and long-term behavior). However, in the first phase, guidance can and should be carried out based first on energy absorption and the reduction in impact loads, which are obtainable by the proposed method.

## 2. Phenomenological Models of the Stress–Strain Behavior of Foams

Not disregarding the theoretical description of the cellular materials extensively described by Gibson and Ashby [1], the first significant empirical law to describe the stress–strain relationship during the compression of a foam material is the relatively simple but effective model introduced by Rusch in 1969 [25]:

(1) $$\sigma \left(\varepsilon \right)=a\varepsilon^{p}+b\varepsilon^{n}$$

It is important to note that, in the Rusch formulation, the stress is related to the true compression strain *ε*. In the case of a uniform strain state, and noting that stress and strain are considered positive in compression (differently to the more usual convention in structural mechanics), it turns out that the relations between the engineering strain *e* and the true strain *ε* values are given by:

(2) $$\varepsilon =-\ln\left(1-e\right)\;↔\;e=1-\exp\left(-\varepsilon \right)$$

The Rusch model is fundamentally empirical and validated through experimental results. It could be rewritten in terms of the simpler engineering strain but, clearly from Equation (2), with a longer and more complex expression.

Many improved descriptions alternative to Equation (1) have been proposed in the literature.

Among the others, this model was introduced by Avalle et al. in [26] to describe the stress–strain characteristics of some plastic foams (EPP, PUR, and a PPA/PS blend):

(3) $$\sigma \left(e\right)=ae^{p}+b{\left(\frac{e}{1-e}\right)}^{n}$$

Another contribution was in [27] by Avalle et al., who tried to improve the approximation in the elastic-plateau phase using the following equation:

(4) $$\sigma \left(e\right)=A\left\{1-\exp\left[-\left(\frac{E}{A}\right)e{\left(1-e\right)}^{n}\right]\right\}+B{\left(\frac{e}{1-e}\right)}^{n}$$

This model was improved by Jeong et al. [28] by adding the influence of the strain rate:

(5) $$\sigma \left(e\right)=\left\{A\left[1-\exp\left(-\left(\frac{E}{A}\right)e{\left(1-e\right)}^{n}\right)\right]+B{\left(\frac{e}{1-e}\right)}^{n}\right\}\left[1+\left(a+be\right)\ln\left(\frac{\dot{\varepsilon }}{{\dot{\varepsilon }}_{0}}\right)\right]$$

An interesting further development, significant because of its generality, as shown in [24], combined a description from [27] and the Rusch model [25], which actually does not properly describe the elastic phase: the derivative of the first term tends to infinity, and this is not physically correct. The model proposed in [24] describes the stress–strain characteristics as follows:

(6) $$\sigma \left(\varepsilon \right)=\sigma_{p}\left[1-\exp\left(-m\varepsilon \right)\right]+\sigma_{s}\varepsilon +\sigma_{D}\varepsilon^{n}$$

where *σ_p_* is the plateau stress level, *σ_s_* the linear hardening slope in the transition phase, *σ_D_* the densification parameter, *m* the linear-plateau transition constant, and *n* the densification exponent.

The strain-rate effect is important and relatively hard to describe: many formulations can be considered such as laws by Cowper–Symonds [29], Johnson–Cook [30], Jones [31], and Jeong [28]. After careful examination of the experimental results and analyses of the different models, it was substantiated that the parameters *σ_p_*, *σ_s_*, and *σ_D_* in Equation (6) can be efficiently adjusted for the strain-rate effect in line with the Cowper–Symonds law, which is:

(7) $$\sigma_{i}=\sigma_{i0}\left[1+{\left(\frac{\dot{\varepsilon }}{{\dot{\varepsilon }}_{0}}\right)}^{p}\right]f_{i}\left(\rho \right)\;\;with\;\;i=p,s,\;D$$

with the parameters, depending on the material type, considering the strain-rate sensitivity ${\dot{\varepsilon }}_{0}$, the reference strain-rate value, and the strain-rate exponent *p*.

**Figure 1:**
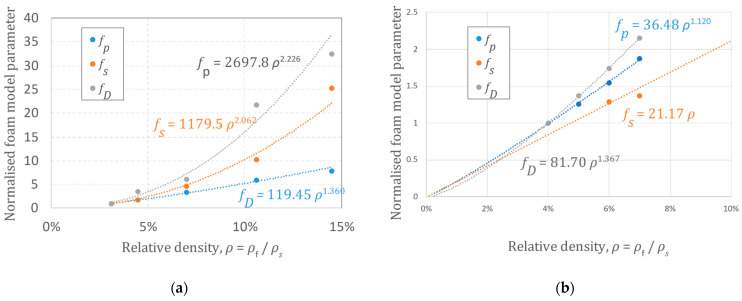
Density parameters as a function of the density: (**a**) expanded polypropylene (EPP, *m* = 50, *n* = 5); (**b**) expanded polystyrene (EPS, *m* = 75, *n* = 5.2).

Finally, the curve parameters *σ_p_*, *σ_s_*, and *σ_D_* of a particular cellular material can be described as functions of a foam’s relative density as shown in [24]:

(8) $$f_{i}\left(\rho \right)=f_{i,0}\;\rho^{n_{i}}\;\;\;with\;\;i=p,s,D$$

where the constants *f_p_*_,0_, *f_s_*_,0_, and *f_D_*_,0_ and exponents *n_p_*, *n_s_*, and *n_D_* can be obtained or estimated for each class of materials. Some results were published in [24]. Figure 1 reports some more experimental results for typical polymeric foams. Shivakumar and Deb [32] also reported results for PP that are comparable in terms of density influence. Care must be taken when considering the influence of density on foam behavior: changing the density affects the cells’ characteristics and behavior. The influence is, therefore, not direct and sometimes not easy to describe. However, as reported in many studies (starting from the well-known mechanical models of Gibson and Ashby), when the density variation is not too large, it can be reasonably approximated by some mathematical function.

## 3. Efficiency Analysis

In [1] the concept of efficiency *E* was introduced:

(9) $$E=\frac{w\left(\sigma \right)}{\sigma }=\frac{\int_{0}^{e}\sigma de}{\sigma }$$

where *w* is the absorbed energy per unit volume at the reached stress level *σ*. It was shown in [2] that the efficiency is exactly a measure of the utilization of the foam material to maximize the energy absorption while minimizing the load. In practice, a certain foam component is efficiently exploited when its stress–strain curve is used to the point of maximum efficiency. If the same component can be made of foams of different densities, the optimal density value is that corresponding to achieving the maximum efficiency for a given input energy.

Maximizing the efficiency *E* can be computed by equating its derivative to zero as follows:

(10a) $$\frac{dE}{de}=\frac{dw}{de}\frac{1}{\sigma }-\frac{w}{\sigma^{2}}\frac{d\sigma }{de}=\sigma \frac{1}{\sigma }-\frac{w}{\sigma^{2}}\frac{d\sigma }{de}=1-\frac{w}{\sigma^{2}}\frac{d\sigma }{de}\;\frac{dE}{de}=0↔\frac{w}{\sigma^{2}}\frac{d\sigma }{de}=1$$

Equation (10a) can also be written in these alternative forms:

(10b) $$\frac{d\sigma }{de}=\frac{\sigma^{2}}{w}=\frac{\sigma }{E}↔\frac{\sigma }{w}=\frac{1}{E}$$

Based on experimental or analytical descriptions of the stress–strain behavior of the cellular material, or materials, of interest, it is relatively easy to obtain—at least numerically—the maximum efficiency. Examples and curves were reported in [2].

It is important to note that working at maximum efficiency is exactly equivalent to operating at the minimum of the cushioning curves. The cushioning curves, introduced several years ago for packaging [33], and used as a design guide in handbooks such as MIL-HDBK-304C [34], are standardized by regulations such as ISO 2248:1985 and ASTM D 1596–14(2021) [35]. The method is quite complex and lengthy, so that many simplified approaches have been proposed as, for example, in [15,20]: this method requires, in fact, many tests under different loading conditions, in terms of material density, loading speed, and impact energy. In the end, however, the method is equivalent to the approach based on efficiency that is presented here: the cushioning curves method aims at minimizing a so-called G-value defined as the ratio between the maximum stress *σ_m_* and the absorbed energy [14]. Of course, the G-value, or better its dimensionless form defined as the cushion factor *C*, equal to the G-value divided by the ratio of the mass falling height h to the sample thickness t, is the inverse of the efficiency *E*: minimizing the G-value or the cushion factor is exactly equivalent to maximizing the efficiency.

## 4. Evaluation of Efficiency Using the New Foam Model

The absorbed energy *w* is computed as the integral of the force times the variation in the length of a constant-section specimen:

(11) $$w\left(\Delta l\right)=\int_{0}^{\Delta l}F\Delta l=\int_{0}^{\Delta l}\sigma A_{0}l_{0}de=V_{o}\int_{0}^{e}\sigma de$$

The absorbed energy per unit volume *w* can be obtained by integration of the stress times the engineering strain. With the current model, the integration can be carried out after a change of variables as follows:

(12) $$w\left(\Delta l\right)=\int_{0}^{\Delta l}F\Delta l=\int_{0}^{\Delta l}\sigma A_{0}l_{0}de=V_{o}\int_{0}^{e}\sigma de$$

After obtaining the derivative of the engineering strain *e* with respect to the true strain *ε*:

(13) $$\frac{de}{d\varepsilon }=\exp\left(-\varepsilon \right)$$

Integration of Equation (6) describing the new model then gives:

(14) $$w\left(e\right)=\int_{0}^{\varepsilon }\sigma \frac{de}{d\varepsilon }d\varepsilon =\sigma_{P}\int_{0}^{\varepsilon }\left[1-\exp\left(-m\varepsilon \right)\right]\exp\left(-\varepsilon \right)d\varepsilon +\;+\sigma_{S}\int_{0}^{\varepsilon }\varepsilon \exp\left(-\varepsilon \right)d\varepsilon +\sigma_{D}\int_{0}^{\varepsilon }\varepsilon^{n}\exp\left(-\varepsilon \right)d\varepsilon =\;=\sigma_{P}\left\{\left[1-\exp\left(-\varepsilon \right)\right]-\frac{1}{m-1}\left[1-\exp\left(-\varepsilon \left(m+1\right)\right)\right]\right\}+\;+\sigma_{S}\left[1-\left(\varepsilon +1\right)\exp\left(-\varepsilon \right)\right]+\sigma_{D}\gamma \left(n+1,\varepsilon \right)=\;=\sigma_{P}\left\{\frac{\left(m-2\right)+\exp\left(-\varepsilon \right)\left[1-m+{\exp\left(-\varepsilon \right)}^{m}\right]}{m-1}\right\}+\;+\sigma_{S}\left[1-\left(\varepsilon +1\right)\exp\left(-\varepsilon \right)\right]+\sigma_{D}\gamma \left(n+1,\varepsilon \right)$$

In terms of the engineering strain *e*:

(15) $$w\left(e\right)=\sigma_{P}\left\{e-\frac{1}{m-1}\left[1-{\left(1-e\right)}^{m+1}\right]\right\}+\;+\sigma_{S}\left[e+\left(1-e\right)\ln\left(1-e\right)\right]+\sigma_{D}\gamma \left(n+1,-\ln\left(1-e\right)\right)$$

where *γ* is the lower incomplete gamma function.

The derivative of the stress–strain curve is expressed by:

(16) $$\frac{d\sigma }{de}=\frac{d\sigma }{d\varepsilon }\frac{d\varepsilon }{de}=\frac{1}{1-e}\frac{d\sigma }{d\varepsilon }=\frac{1}{1-e}\left\{{m\sigma }_{p}\exp\left(-m\varepsilon \right)+\sigma_{s}+n\sigma_{D}\varepsilon^{n-1}\right\}=\;=\frac{1}{1-e}\left\{{m\sigma }_{p}\exp\left(m\ln\left(1-e\right)\right)+\sigma_{s}+n\sigma_{D}{\left[-\ln\left(1-e\right)\right]}^{n-1}\right\}$$

Equation (10a) does not have an explicit solution, but it can be solved numerically. Since:

(17) $$\frac{d\sigma }{de}=\frac{\sigma^{2}}{w}\;w\left(e\right)\frac{d\sigma }{de}-\sigma^{2}=0$$

By noting that:

(18) $$\sigma \left(e\right)=\sigma_{p}\left[1-{\left(1-e\right)}^{m}\right]-\sigma_{s}\ln\left(1-e\right)+\sigma_{D}\left[-{\ln\left(1-e\right)}^{n}\right]$$

Equation (18) can be explicitly stated as:

(19) $$\left[\sigma_{P}\left\{e-\frac{1}{m-1}\left[1-{\left(1-e\right)}^{m+1}\right]\right\}+\sigma_{S}\left[e+\left(1-e\right)\ln\left(1-e\right)\right]+\sigma_{D}\gamma \left(n+1,-\ln\left(1-e\right)\right)\right]\times \;\times \left[\frac{1}{1-e}\left\{m\sigma_{p}{\left(1-e\right)}^{m}+\sigma_{s}+n\sigma_{D}{\left[-\ln\left(1-e\right)\right]}^{n-1}\right\}\right]=\;={\left\{\sigma_{p}\left[1-{\left(1-e\right)}^{m}\right]-\sigma_{s}\ln\left(1-e\right)+\sigma_{D}\left[-{\ln\left(1-e\right)}^{n}\right]\right\}}^{2}$$

To simplify the numerical solution, it is useful to note that the quantities in Equation (19) are all related to the foam parameters *σ_p_*, *σ_s_*, and *σ_D_*. Then, expressions (17)–(19) can be expressed as:

(20) $$w\left(e\right)=\sigma_{P}f_{P}\left(e\right)+\sigma_{s}f_{s}\left(\epsilon \right)+\sigma_{D}f_{D}\left(e\right)\;\sigma \left(e\right)=\sigma_{P}f_{P}^{\prime }\left(e\right)+\sigma_{s}f_{s}^{\prime }\left(e\right)+\sigma_{D}f_{D}^{\prime }\left(e\right)\;\frac{d\sigma }{de}=\sigma_{P}f_{p}^{\prime \prime }\left(e\right)+\sigma_{s}f_{s}^{\prime \prime }\left(e\right)+\sigma_{D}f_{D}^{\prime \prime }\left(e\right)$$

So that, by rewriting Equation (17) as follows, the solution can be numerically evaluated:

(21) $$\frac{\frac{d\sigma }{de}}{\sigma }=\frac{\sigma }{w}\;\frac{\sigma_{P}f_{p}^{\prime \prime }\left(e\right)+\sigma_{s}f_{s}^{\prime \prime }\left(e\right)+\sigma_{D}f_{D}^{\prime \prime }\left(e\right)}{\sigma_{P}f_{P}^{\prime }\left(e\right)+\sigma_{s}f_{s}^{\prime }\left(e\right)+\sigma_{D}f_{D}^{\prime }\left(e\right)}=\frac{\sigma_{P}f_{P}^{\prime }\left(e\right)+\sigma_{s}f_{s}^{\prime }\left(e\right)+\sigma_{D}f_{D}^{\prime }\left(e\right)}{\sigma_{P}f_{P}\left(e\right)+\sigma_{s}f_{s}\left(e\right)+\sigma_{D}f_{D}\left(e\right)}$$

Of course, determination of the maxima can be made directly on the experimental curves but with a longer processing of the experimental data and without the generality obtained from an accurate predictive model.

## 5. Simplified Selection Charts with the Foam Model

The analysis of the efficiency curves to optimize the selection of a foam for a particular application, as is the case with the cushioning method, starts from the experimental characterization of several samples (or, in principle, from the predictions of a theoretical model such as that by Gibson–Ashby). This will lead to the construction of the selection diagrams as in the examples reported in [2]. This is a relatively simple task, and even simplified further if the material model is identified including the influence of the material properties and other factors. However, the points in the curves of the selection diagrams are to be obtained numerically since, as shown in the previous section, an explicit solution is not available (at least with the present model: with simpler, yet less accurate models, it could theoretically be feasible).

**Figure 2:**
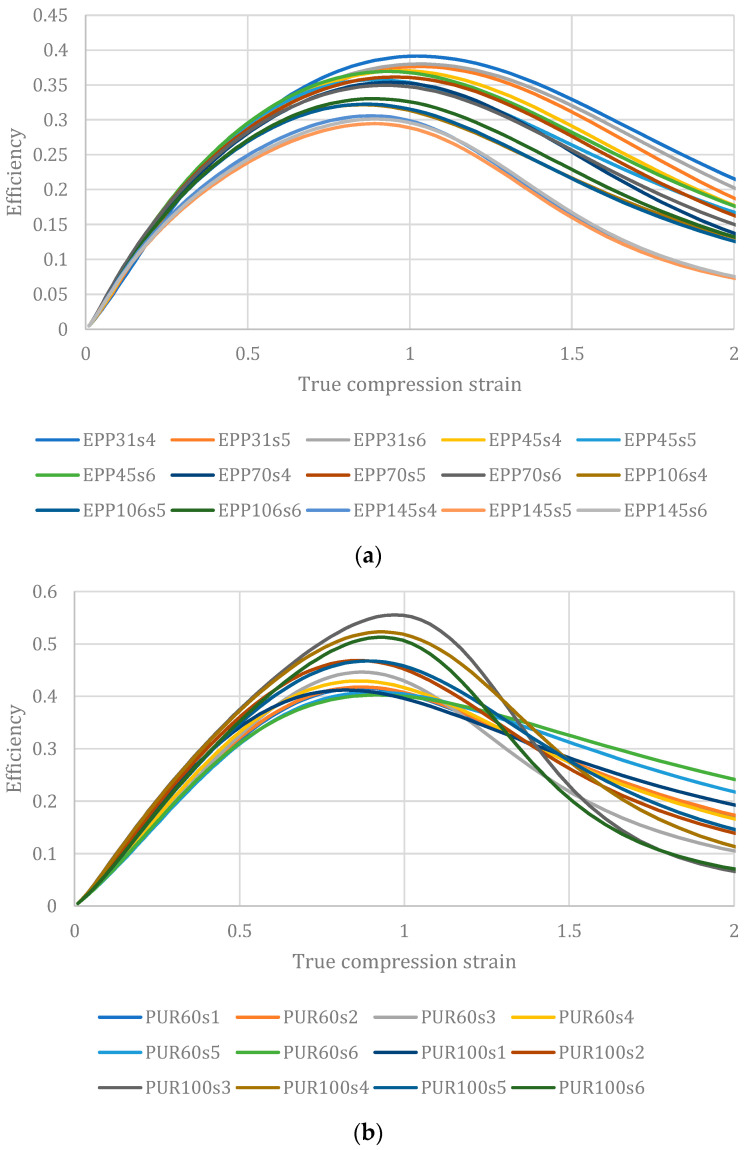

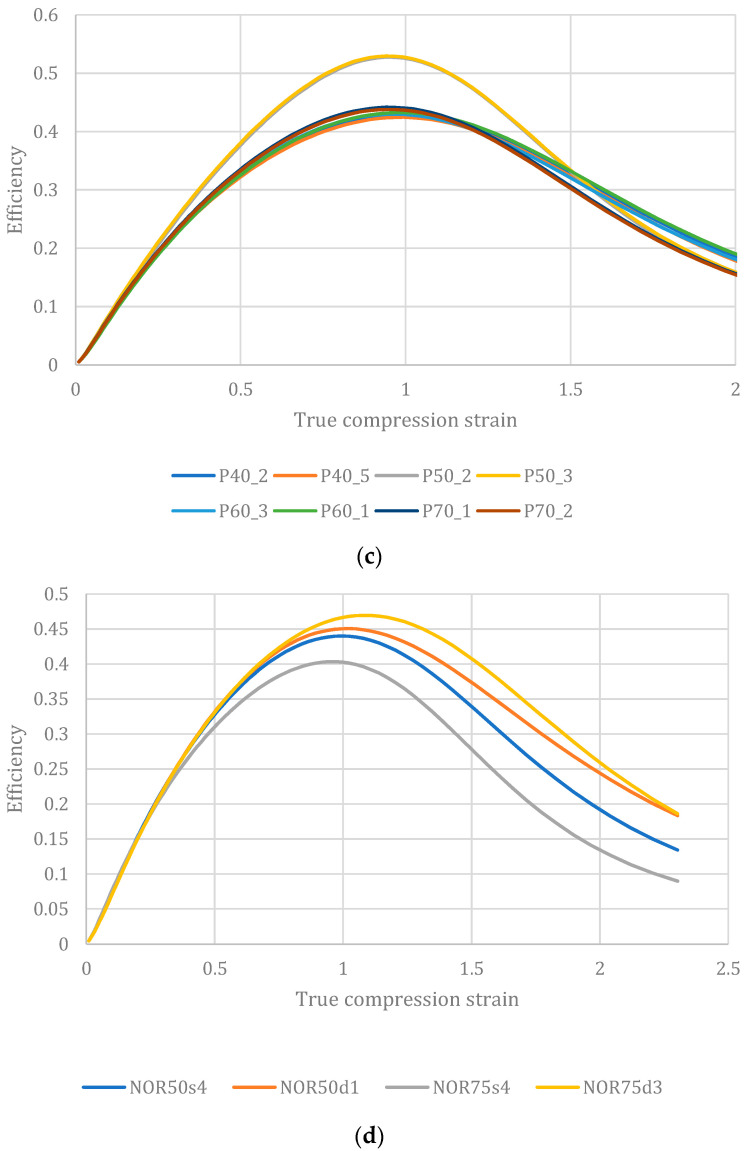
Efficiency diagrams for four different polymeric foams of various values of density (random selection from numerous samples): (**a**) expanded Polypropylene, EPP; (**b**) expanded Rigid Polyurethane, PUR; (**c**) expanded Polystyrene, EPS; (**d**) commercial polystyrene–PMA blend, Noryl GTX^®^.

However, a serendipitous observation carried out on several polymeric foams examined in the previously reported papers by the author and co-authors [2,27], revealed an interesting outcome. Those results, in terms of efficiency versus true strain, are reported in Figure 2.

For all examined materials, even of different solid materials with very different natures and behaviors (some have a large plateau like PUR, in others this is less marked) and at the densities examined, all the materials exhibit maximum efficiency at the unit value of the true compression strain. Incidentally, a similarity is confirmed by the results of other authors with similar materials such as, for example, in [32] for rigid PU, ref. [36] for EPS, an refs. [37,38] for aluminum foams. Of course, the precise value is never exactly one but assuming this value in the described equations makes it possible to greatly simplify their expression and even to quickly obtain the selection diagrams. This, at least in the preliminary design stages, can greatly accelerate material selection for safety and packaging applications once the foam properties are obtained.

By substituting the unit value of the true compression strain, the following expressions for compression stress and absorbed energy are then obtained:

(22) $$\sigma \left(\rho ;\varepsilon =1\right)=\sigma_{p}\left(\rho \right)\left[1-\exp\left(-m\right)\right]+\sigma_{s}\left(\rho \right)+\sigma_{D}\left(\rho \right)\;w\left(\rho ;\varepsilon =1\right)=\sigma_{P}\left(\rho \right)\left\{\frac{\left(m-2\right)+\exp\left(-1\right)\left[1-m+\exp\left(-m\right)\right]}{m-1}\right\}+\;+\sigma_{S}\left(\rho \right)\left[1-2\exp\left(-1\right)\right]+\sigma_{D}\left(\rho \right)\gamma \left(n+1,1\right)$$

Note: the expression *exp*(−1) which is, of course, the inverse of Euler’s number is kept in this form and not using the usual name of this constant to avoid confusion with the engineering strain which is defined by the same letter *e*.

**Figure 3:**
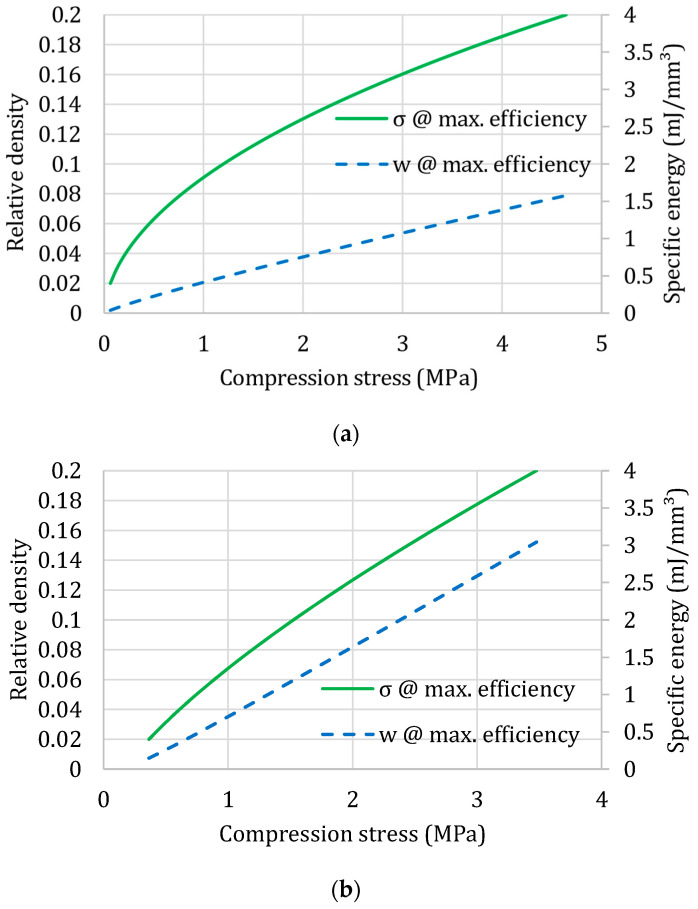

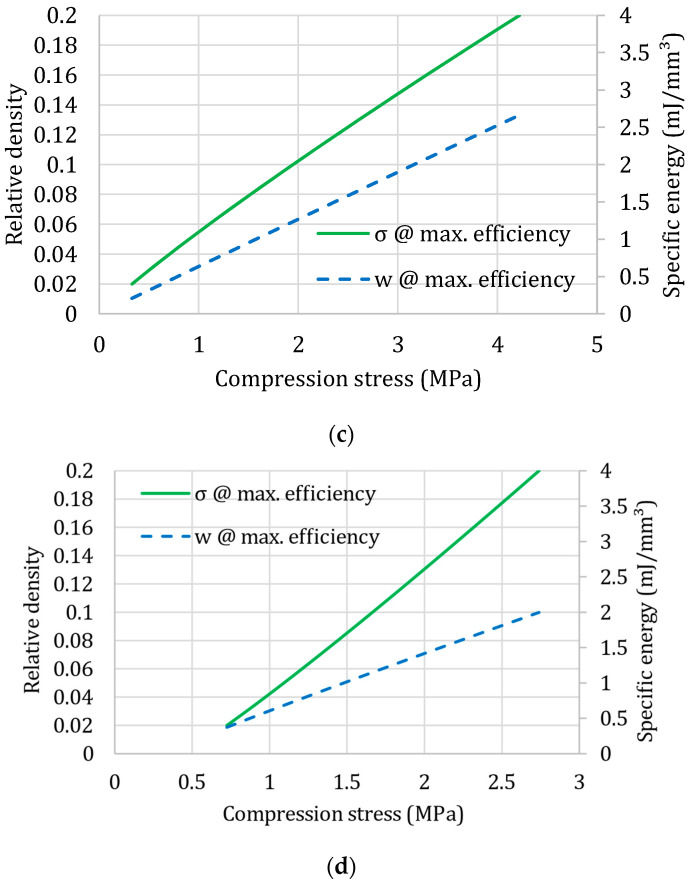
Selection diagrams for four different polymeric foams: (**a**) expanded Polypropylene, EPP; (**b**) expanded Rigid Polyurethane, PUR; (**c**) expanded Polystyrene, EPS; (**d**) commercial polystyrene–PMA blend, Noryl GTX^®^.

Figure 3 includes the four simplified selection diagrams obtained, based on the identification of the foam constants and exponents, for the four materials examined before as in Figure 2.

The reported results are for quasi-static conditions: of course, it is possible to generate the same diagrams for different loading rates—the approximation with the material law of Equations (6)–(8) was demonstrated as valid including other influencing factors in [24]. So, the selection diagrams can be drawn, for example, for different strain rates as shown in [2].

The process to generate the selection diagrams for another class of expanded materials will be, in synthesis:

1. If the expanded material properties are not known, perform a compression test at several values of density in the typical range of interest, and—when required—different loading conditions.
2. Identify the parameters of the law expressed by Equations (6)–(8): this can be completed with any non-linear numerical fitting tool.
3. Check that the assumption that the maximum efficiency at the unit value of the true compression strain is really applicable.
4. Draw the selection diagrams in the measured range of relative densities by using the expression of Equation (22).

Of course, if the condition of point 3 is not verified, the simplified approach described here cannot be used, and the selection diagrams must be obtained by analyzing the individual results using efficiency diagrams to obtain the maxima. If the maxima occur at a different constant value of the true compression strain, simplified expressions equivalent to that of Equation (22) can be obtained.

As usual, extrapolation outside the measured range of densities is possible, but by taking care not to exceed reasonable values: some experience on the behavior of the specific material is useful if not necessary.

The proposed method is simplified and introductory: in general, it considers only the first, monotonical, loading of the foam. Therefore, its application is restricted to those applications where energy is absorbed in a first impact. This is, of course, a relatively strong limitation; however, it does not exclude several relevant applications: in the case of a crash, in some packaging applications and other safety problems, the first impact determines most of the involved phenomena and energy management. In those cases, loading is practically isothermal due to the high strain rate; so, thermal effects are limited. In other cases, involving low velocity impacts, and repeated impacts, the method is not useful if not used solely for a first rough evaluation.

Additionally, density is considered to be the primary foam parameter; indeed, it is the most impactful factor, as shown by the fundamental theoretical approach by Gibson and Ashby [1]. However, real cellular materials, especially polymer-based materials, are largely sensitive to other influencing factors (such as temperature, strain rate, cell structure and properties, etc.) and are sometimes difficult to correctly describe even using complex mathematical models. Some of these factors can be modeled, as in Equations (7) and (8), and included in this method. However, not all behavior can be completely modeled, and care must be taken that the adopted models are valid within the range of application and validity of the problem.

## 6. Application Example

As an example of the simplicity and advantage of the proposed method, the initial guess regarding the foam density in a pedestrian protection application is shown.

The problem is related to legform impact as stated by the European Regulation (EC) No 78/2009, amending Directive 2007/46/EC. This standard method was also adopted by EuroNCAP in 2015 [39]. In that method, leg protection is assessed by projecting a 7.4 kg legform travelling at a 40 km/h speed onto the front bumper. Leg protection is then evaluated by measuring the dynamic and kinematic response (such as tibia acceleration, impact forces, etc.). The design of the bumper and vehicle front end have a decisive impact on the responses and the possible level of damage to the leg of the impacted pedestrian [40]. A simple model of the bumper can be reduced to a block of rigid foam absorbing the impact energy of the legform. Optimization of the foam, in particular via optimal density selection, can be performed using the proposed method to define an initial design.

In this example, expanded polypropylene (EPP) is chosen. EPP is largely used as a filler for car bumpers due to its characteristics of stability, machinability, resistance to environmental factors, cost, and—last but not least—being a material widely used in car components; thus, contributing to the reduction in the number of materials for recycling and reuse. In this simple example, the foam is used alone to absorb the initial impact energy equal to 5.92 kJ. The chosen foam block is a cube with 40 mm sides; therefore, the impact energy to be absorbed is 92.5 × 10^−3^ J/mm^3^.

Based on the results of three tests on EPP specimens with densities of 31, 70, and 145 g/dm^3^ [2,26], fitting Equation (6) with the experimental data provides the model parameters reported in Table 1. Table 1 also includes the optimal parameters from Equation (22) for this material.

**Figure 4:**
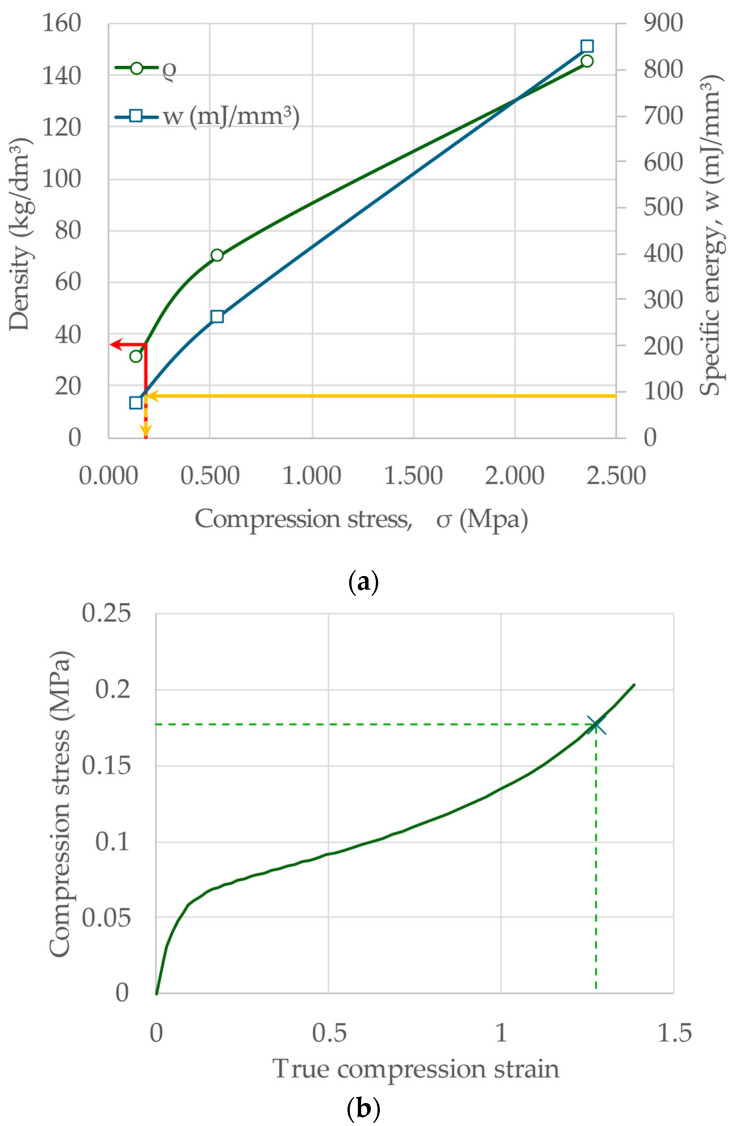
Simplified selection diagram of EPP foams: (**a**) selection diagram and optimal point for the bumper/leg protection application: selection usually starts from specific energy value (left axis, yellow line) horizontally to the respective curve (in blue) to obtain the compression stress value (on abscissa), from this point vertically up to the density curve (red line) to get the optimal density value; (**b**) stress–strain characteristic of the selected foam with the optimal point.

Based on these parameters, an approximate selection diagram for this family of EPP can be drawn (Figure 4) and, with the specific energy input reported above, the optimal suggested density for this application is around 36 g/dm^3^, with a maximum compression stress of 18 MPa. The input is the specific energy on the right vertical axis. The optimal compression stress is determined in abscissa; from this value, vertically, the density is then identified.

Of course, the construction of the diagram is largely oversimplified, but a more refined version of the diagram can be obtained with more data (as from Figure 2a in [2]). The basic forecast determined by using a reduced set of data is, however, not without useability since it is based on a fitting with a defined law (in Equations (6) and (22)).

## 7. Conclusions

The design of energy absorbing structures made of cellular materials, at least in a preliminary phase, can be made by using simplified methods. Among these methods, cushioning curves have been extensively used for packaging applications; whereas, for safety purposes, efficiency curves are more common. In practice, the two methods are equivalent and the selection of the optimal material, that is the foam density given a certain energy and the size of the energy absorbing component, is based on searching for the minimum or maximum of a parameter representing the most efficient solution. The optimal solution gives the best performance in terms of maximum absorbed energy while minimizing the load generated on the protected good or system.

Thanks to modeling the material’s behavior, and the influence of internal and external parameters, the application of these methods can be greatly simplified or, at least, shortened by reducing both the number of tests on foam samples and the analysis of the test results. In fact, the basis of the mentioned methods is a series of experimental tests, usually compression tests, on samples of different densities and, possibly, various loading rates, temperatures, etc. Those experimental results can be used to derive curves in selection diagrams aiming to quickly and easily help in the selection of the optimal expanded material. Starting the design of an energy-absorbing device from the simplified models described in this paper can give the impression of being simplistic, as discussed in [21]. However, in this same paper, the use of simplified models based on simple compression tests is justified in the preliminary design phases. And, for this purpose, such simplified approaches have been effectively used in the design of several applications such as protective devices in sports applications [19], such as helmets, but also devices that are not properly foams but are equivalently energy absorbing, like climbing or worker security ropes [18].

The present work, based on experimental results and a model by the author and other co-authors, proposes a simplified method to build these selection diagrams based on a serendipitous observation of the experimental results of many materials with different natures. Since it appears that the maximum efficiency occurs at an almost fixed value of strain (a result apparently found in other cases with different materials [36,37,38]), the selection curves can be drawn quickly with simplified mathematical expressions. This can shorten and simplify the selection process. Of course, the standard process of building the selection diagrams can be used, even only for a cross-check, and a detailed modeling of the energy absorbing component will probably follow, especially in applications with critical safety issues. However, the simplified method can be a useful basic tool to start with.

## Figures and Tables

**Table 1:** Material parameters in Equation (6) and optimal parameters of Equation (22) for EPP material samples.

Sample	Foam Density (g/dm^3^)	*σ_p_*	*σ_s_*	*σ_D_*	*m*	*n*	Σ (MPa)	*w* (mJ/mm^3^)
EPP31s4	31	0.0602	0.0616	0.0127	21.5	4.62	0.135	0.076
EPP70s5	70	0.1791	0.3016	0.0579	68.7	5.11	0.539	0.261
EPP145s6	145	0.3988	1.6589	0.3047	55.7	6.79	2.363	0.849

## Data Availability

The raw data supporting the conclusions of this article will be made available by the authors on request.
